# Long read sequencing reveals transgene concatemerization and vector sequences integration following AAV-driven electroporation of CRISPR RNP complexes in mouse zygotes

**DOI:** 10.3389/fgeed.2025.1582097

**Published:** 2025-06-04

**Authors:** Muhammad W. Luqman, Piroon Jenjaroenpun, Jessica Spathos, Nikhil Shingte, Mitchell Cummins, Pattaraporn Nimsamer, Lars M. Ittner, Thidathip Wongsurawat, Fabien Delerue

**Affiliations:** ^1^ Dementia Research Centre, Macquarie Medical School, Faculty of Medicine, Health and Human Sciences, Macquarie University, Sydney, NSW, Australia; ^2^ Khyber Medical University, Institute of Medical Sciences, Kohat, Pakistan; ^3^ Division of Medical Bioinformatics, Research Department, Faculty of Medicine Siriraj Hospital, Mahidol University, Bangkok, Thailand; ^4^ FOXG1 Research Foundation, New York, NY, United States; ^5^ School of Biotechnology and Biomolecular Sciences, The University of New South Wales, Sydney, NSW, Australia

**Keywords:** long read sequencing (LRS), CRISPR, adeno-associated-virus (AAV), mice, zygotes, concatemers

## Abstract

Over the last decade CRISPR gene editing has been successfully used to streamline the generation of animal models for biomedical research purposes. However, one limitation to its use is the potential occurrence of on-target mutations that may be detrimental or otherwise unintended. These bystander mutations are often undetected using conventional genotyping methods. The use of Adeno-Associated Viruses (AAVs) to bring donor templates in zygotes is currently being deployed by transgenic cores around the world to generate knock-ins with large transgenes (i.e., 1–4 kb payloads). Thanks to a high level of efficiency and the relative ease to establish this technique, it recently became a method of choice for transgenic laboratories. However, a thorough analysis of the editing outcomes following this method is yet to be developed. To this end, we generated three different types of integration using AAVs in two different murine genes (i.e., *Ace2* and *Foxg1*) and employed Oxford Nanopore Technologies long read sequencing to analyze the outcomes. Using a workflow that includes Cas9 enrichment and adaptive sampling, we showed that unintended on-target mutations, including duplication events and integration of viral sequences (sometimes reported using other workflows) can occur when using AAVs. This work highlights the importance of in-depth validation of the mutant lines generated and informs the uptake of this new method.

## Introduction

Genetically modified (GM) animals, particularly mice, are powerful models to understand the mechanisms underlying physiological processes and human disorders. They are also invaluable tools to develop and test novel treatment strategies.

Transgenic laboratories around the world generate these models for biomedical research purposes, using either microinjection techniques (Delerue and Ittner, 2017), or more recently electroporation of fertilized zygotes (Qin et al., 2015; Kaneko and Mashimo, 2015). Electroporation is less challenging than microinjection and allows for high-throughput transformation of zygotes, whereas microinjection requires manipulation of zygotes one at a time. Moreover, survival and development rates are comparatively higher because electroporation is less invasive and damaging to the embryos than microinjection (Kaneko and Mashimo, 2015). As such, electroporation of zygotes is widely used to generate knockouts (KO) and small nucleotides changes, such as point mutations or base pair exchanges. We, and others, generated such mouse models using electroporation of one-cell embryos (Klugmann et al., 2022; Morey et al., 2022).

However, electroporation remains largely inefficient at driving the targeted integration of large transgenes to generate knock-in (KI) mouse lines, presumably because the zona pellucida (thick glycoprotein membrane protecting the embryos at the preimplantation stages) prevents the shuttling of these large transgenes inside the embryos. To the best of our knowledge, there has been no report to date of a successful transformation of double stranded DNA (e.g., plasmid or transgene) using electroporation of such zygotes, and the largest insertion reported so far by electroporation of single-stranded oligo is 1 kb in length (Miyasaka et al., 2018).

Therefore, a new method based on infection of zygotes with Adeno-Associated Viruses (AAVs) to bring the donor template inside the zygotes, coupled with electroporation of CRISPR ribonucleoprotein (RNP) complexes to induce Homology Directed Repair (HDR) has recently been developed (Yoon et al., 2018; Mizuno et al., 2018; Chen et al., 2019). Such method, coined “CRISPR-READI” shows high level of efficiency (up to 100% in our hands) and is relatively easy to implement in transgenic laboratories. Romeo *et al.* showed that AAVs could diffuse through the zona pellucida (Romeo et al., 2020), while the Rivera-Perez lab demonstrated that the transfection efficiency varies depending on the serotype used, serotype 6 having one of the highest levels of transduction in mouse zygotes (Yoon et al., 2018).

Over the last decade CRISPR gene editing has been extensively used to generate GM mice, however, one limitation to its use is the potential occurrence of on-target mutations that are detrimental or otherwise unintended. These bystander mutations are typically undetected using conventional genotyping (i.e., PCR) and routine (i.e., Sanger) sequencing (Simkin et al., 2022; Sailer et al., 2021). As such, an in-depth analysis of the editing outcomes is highly recommended (Lintott and Nutter, 2023) to ensure that GM animals are validated before extensive breeding.

Recently, Oxford Nanopore Technologies (ONT^©^) Long Read Sequencing (LRS) has been used in mice to identify the insertion site of randomly integrated transgenes (Bryant et al., 2023), targeted insertions (McCabe et al., 2024), and to confirm integration following recombinase-mediated cassette exchange (RMCE) (Low et al., 2022). However, to the best of our knowledge, LRS has not yet been used to perform quality control (QC) following AAV-driven gene editing in zygotes. To this end, we performed CRISPR-READI to target two murine genes (i.e., *Ace2* and *Foxg1*) with transgenes of various sizes. We then analyzed these knock-ins using the Oxford Nanopore Technologies (ONT^©^) MinION Mk1c and/or GridION, following a Cas9-based enrichment method that we previously applied to cells (Wongsurawat et al., 2020). Using this method, we identified instances of concatemerization with partial AAV vector sequences integration (particularly Inverted Terminal Repeat sequences - ITR) in two out of five (40%) mouse lines generated.

## Results

In our workflow, following PCR genotyping of the Founder mice, Long Read Sequencing was performed either at the F1 or the F2 generation (see Supplementary Figure S1 for the identification of each selected mouse in this study).

### Generation of KI mouse lines

We performed AAV-driven gene editing on two different murine genes: the angiotensin I converting enzyme 2 (i.e., *Ace2*) and the forkhead box G1 (i.e., *Foxg1*) to insert three different transgenes.

**FIGURE 1:**
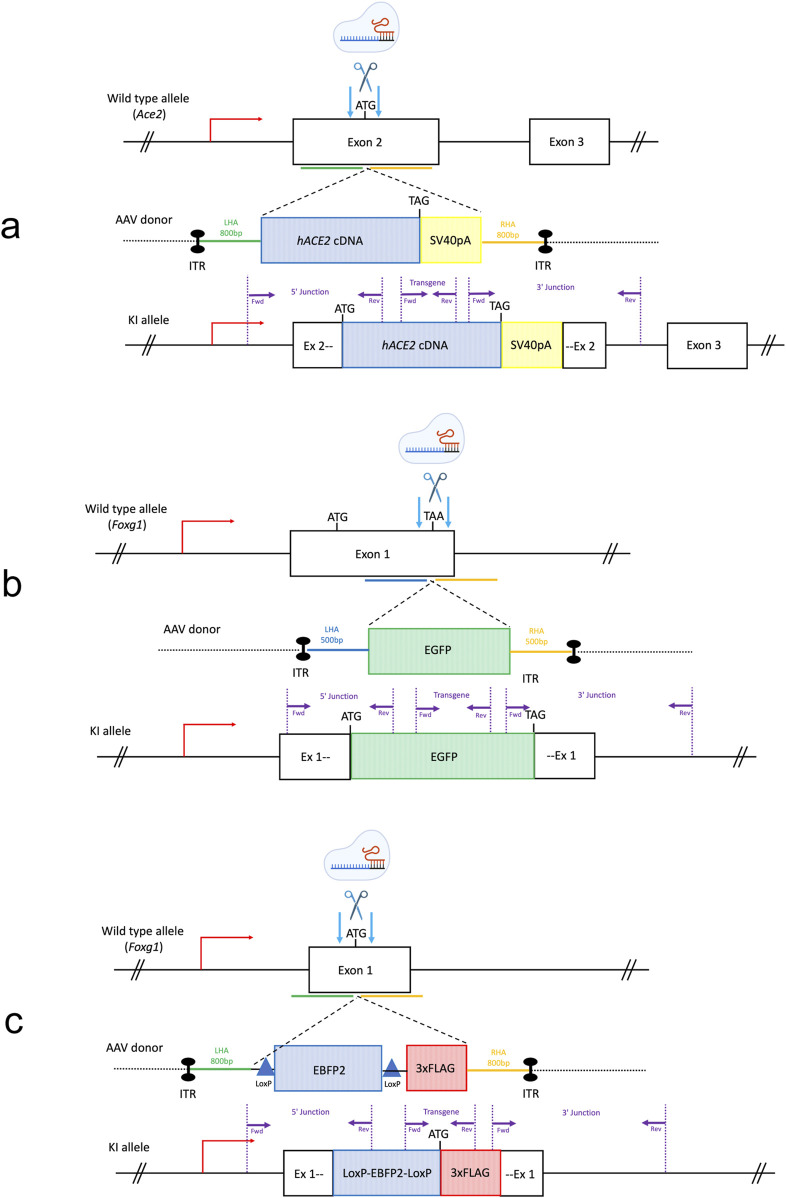
Generation of KI mouse lines by AAV-driven gene editing. The gene editing strategy for each knock-in (KI) line is illustrated. **(a)** The start codon of the murine *Ace2* gene was targeted for Homology Directed Repair (HDR) with a donor template carrying the *hACE2* coding DNA sequence upstream of a PolyA. **(b)** The stop codon of the mouse *Foxg1* gene was targeted for HDR with an EGFP sequence at the c-terminus. **(c)** The start codon of the murine *Foxg1* gene was targeted to insert a stop cassette and a triple FLAG tag. LHA = left homology arm, RHA = right homology arm, ITR = inverted terminal repeat sequence, *hACE2* = human ACE2 coding sequence, SV40 pA = simian virus 40 polyadenylation signal, EGFP = Enhanced Green Fluorescence Protein sequence, EBFP2 = Enhanced Blue Fluorescent Protein sequence, 3xFLAG = triple FLAG tag, LSL = Lox-Stop-Lox. Purple arrows: location of the genotyping primers used in this study for the transgene-specific and the junction PCRs. Light blue arrows: endonucleases-induced double strand breaks.

First, we targeted the start codon of the murine *Ace2* gene and inserted the human *ACE2* (*hACE2*) coding sequence upstream of a polyadenylation signal (SV40 pA) (Figure 1a), using a combination of CRISPR/Cas9 and TALEN nucleases (no suitable sgRNA could be identified to target this genomic sequence, see *Methods*).

We next targeted the stop codon of the endogenous *Foxg1* gene to insert an Enhanced Green Fluorescent Protein (EGFP) sequence in frame (Figure 1b). Finally, we also targeted the start codon of the *Foxg1* gene to generate a conditional KI by inserting a Lox-Stop-Lox (LSL) cassette (made of an EBFP2 coding sequence flanked by two LoxP sites) upstream of a triple FLAG sequence (Figure 1c).

The size of the transgenes ranged from ∼700 bp to 2.5 kb, and 5 h infection with high titers of recombinant AAV6 was performed for all KIs (a summary of the strategy used for the three KI approaches is detailed in Table 1).

**TABLE 1:** CRISPR-READI strategy to generate the KI mouse lines. Details of the transgenes and editing specifications used to generate the mouse lines by AAV-driven gene editing. bp = base pairs, GC/μL = genome-copy per microliter, ng/μL = nanogram per microliter.

Targeted gene	ACE2	FOXG1	FOXG1
Mouse line	hACE2	Foxg1-EGFP	Foxg1 cKI
Transgene	hACE2	EGFP-pA	LSL-3xFLAG
Size of transgene	2536 bp	717 bp	1933 bp
Homology Arms	800 bp	500 bp	800 bp
Serotype	AAV6	AAV6	AAV6
Incubation	5 h	5 h	5 h
Titer	3.75 × 10E8 GC/μL	1.96 × 10E8 GC/μL	1.62 × 10E8 GC/μL
S.p.Cas9	200 ng/μL	200 ng/μL	200 ng/μL
Editor 1	sgRNA: 400 ng/μL	sgRNA: 100 ng/μL	sgRNA: 100 ng/μL
Editor 2	TALEN: 100 ng/μL	sgRNA: 100 ng/μL	sgRNA: 100 ng/μL

### PCR genotyping of the KI mice does not identify any illegitimate mutation

#### *hACE2* KI mice

**FIGURE 2:**
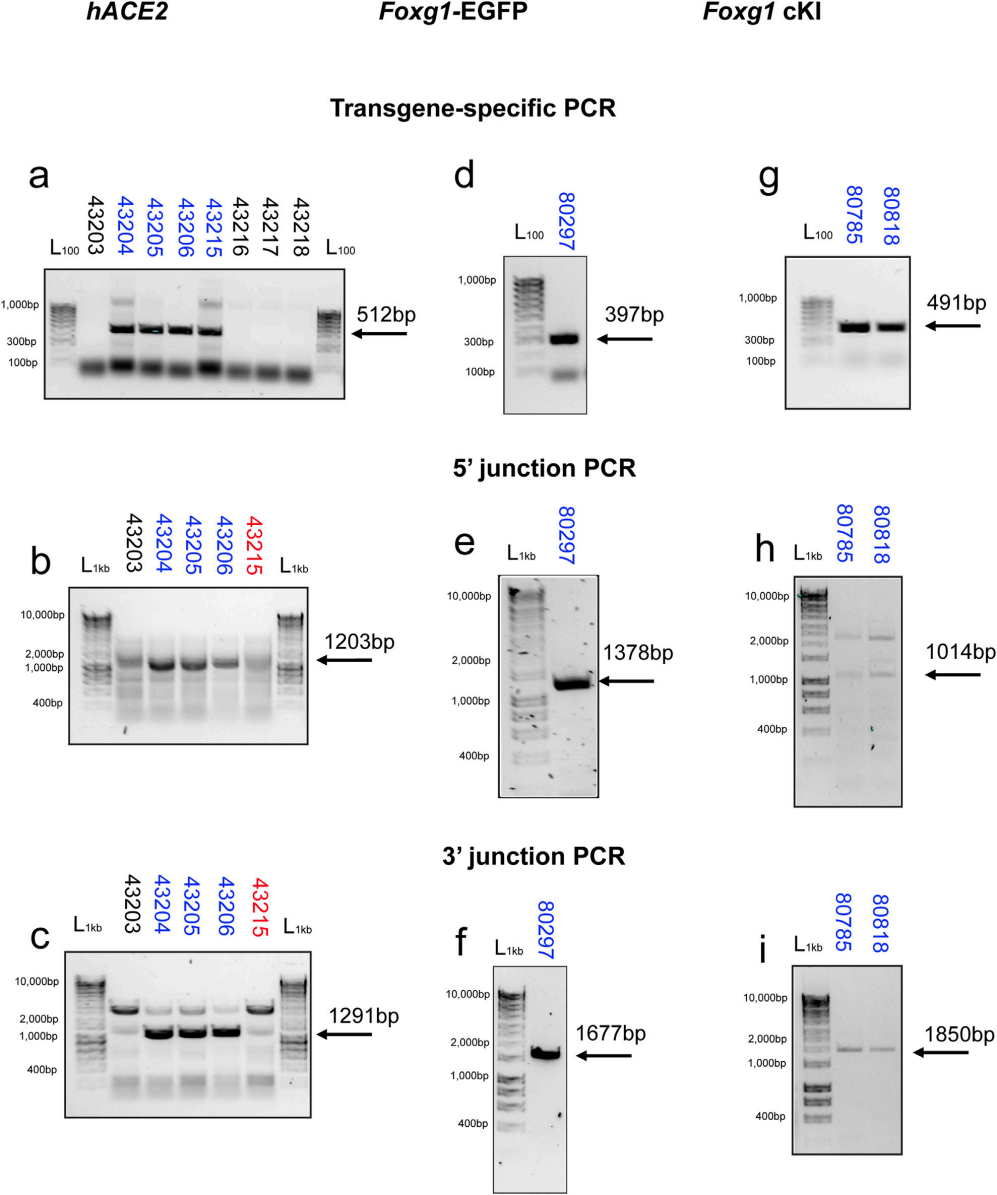
PCR Genotyping outcome for the KI mouse lines. Illustrative cropped gel electrophoresis for the three KI mouse lines generated by CRISPR-READI. **(a)** Transgene-specific genotyping of the *hACE2* founders (G0) showed that 4 out of 8 pups generated (50%) carried the hACE2 transgene. Targeted integration of the hACE2 transgene was confirmed by 5’ **(b)** and 3’ **(c)** junction PCRs. Founder #43215 may carry the AAV template as an episome or be randomly integrated. **(d)** Transgene-specific genotyping of the selected *Foxg1*-EGFP line (G1 generation). Targeted integration for the *Foxg1*-EGFP line was confirmed by 5’ **(e)** and 3’ **(f)** junction PCRs. **(g)** Transgene-specific genotyping of the selected *Foxg1* conditional KI (*Foxg1* cKI) line (G1 generation). Targeted integration for the *Foxg1* cKI line was confirmed by 5’ **(h)** and 3’ **(i)** junction PCRs. L_1kb_: Ladder (Bioline, HyperLadder 1 kb); L_100_: Ladder (Bioline, HyperLadder 100 bp).

To identify potential founders, we first ran a transgene-specific PCR. Out of eight pups, four (50%) carried the transgene (Figure 2a). To validate the insertion of the transgene at the endogenous *Ace2* locus, we then performed 5′ and 3′ junction PCRs (Figures 2b,c). Out of four potential founders, three carried the transgene at the insertion site. The fourth one (#43215) may display random insertion or episomal presence of the transgene. Although not always completely specific (e.g., Figure 2a presents with extra bands), the PCR genotyping did not reveal any apparent illegitimate event at the targeted site. We next selected two founders (#43204 and 43205) to establish the colonies.

#### *Foxg1* KI mice

Similar to the *hACE2* KI mice, PCRs identified several candidate *Foxg1* KI Founders. Indeed, transgene-specific PCR identified sixteen potential founders out of 41 live pups (39%) for the *Foxg1*-EGFP; and three potential founders out of 18 live pups (17%) for the *Foxg1* cKI, respectively.

We selected one *Foxg1*-EGFP founder and two *Foxg1* cKI founders and bred them with wildtype C57BL/6J mice to establish the colonies. We performed transgene-specific and junction PCRs on F1 mice and observed a normal genotyping profile with expected band sizes for all *Foxg1* KI mice (Figures 2d–i). The sequence of all primers used in this study can be found in Supplementary Table S1.

### Cas9 enrichment generates adequate level of coverage

**FIGURE 3:**
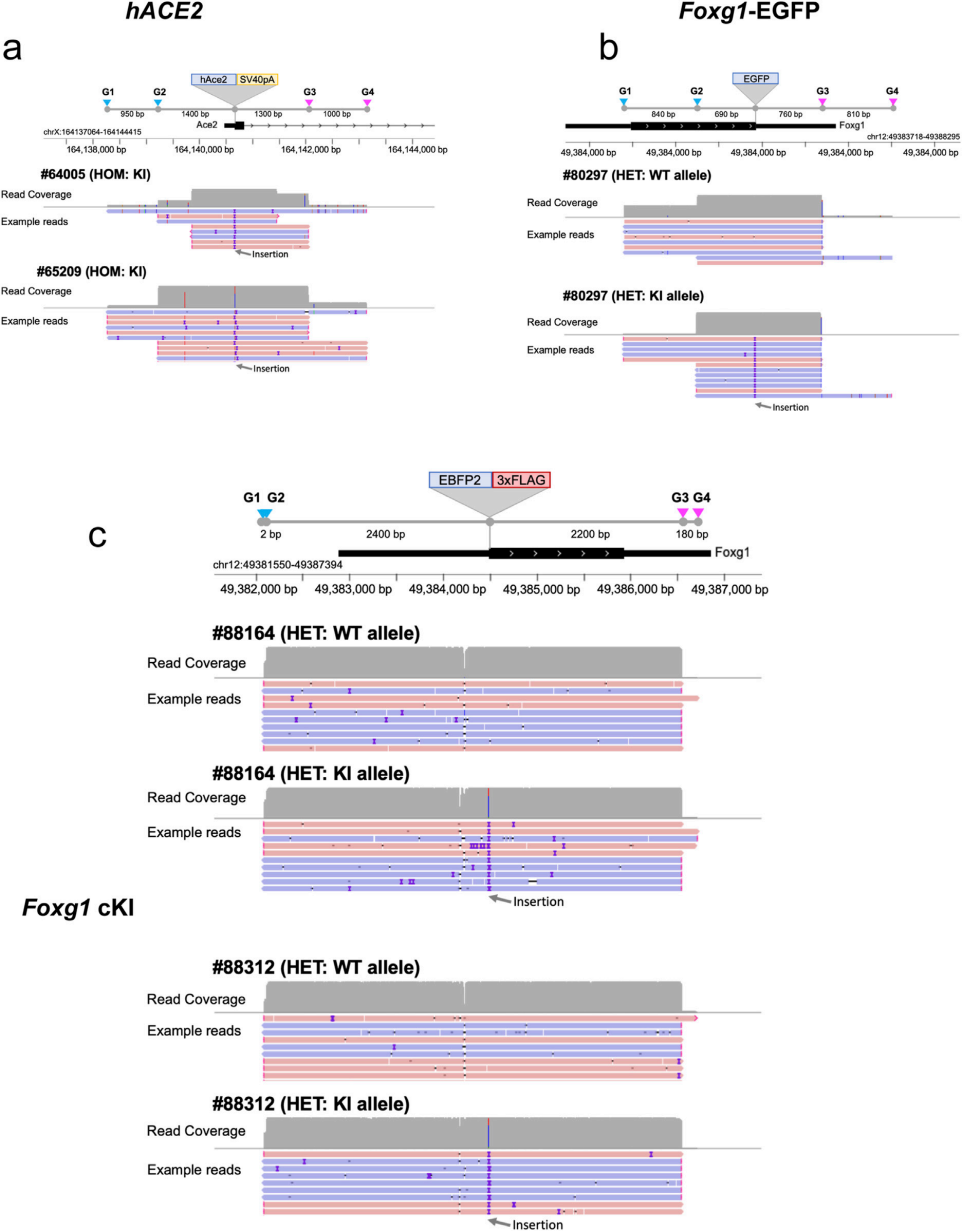
Cas9 enrichment strategy and resulting read depth for each KI. Illustration of the Cas9-based enrichment (nCATs method) of genomic DNA using four sgRNA and resulting read coverage. **(a)** Enrichment of the murine *Ace2* targeted region using four sgRNA encompassing the insertion site of the hACE2 transgene. Example of reads (pink = 5’ to 3’ reads, blue = 3’ to 5’ reads, purple = mismatch) for each homozygous *hACE2* mouse tested (#64005 and #65209). **(b)** Enrichment of the murine *Foxg1* targeted region using four sgRNA encompassing the insertion site of the EGFP transgene. Example of reads for the heterozygous *Foxg1*-EGFP mouse tested (#80297). **(c)** Enrichment of the murine *Foxg1* targeted region using four sgRNA encompassing the insertion site of the EBFP2-3xFLAG transgene. Example of reads for the two heterozygous *Foxg1* cKI mouse tested (#88164 and #88312). G1-G4 = sgRNA1 to sgRNA4, WT = wildtype, KI = knock-in.

We performed genomic enrichment for five mice, two homozygous (#64005 from Founder #43205 and #65209 from Founder #43204) and three heterozygous (#80297, #88164 and #88312), and designed four sgRNAs for each locus (see *Methods*) to enrich the genomic regions of interest (ROI), following the nanopore Cas9-targeted sequencing (nCATS) method (Gilpatrick et al., 2020). The enrichment targeting a ∼3–5 kb ROI centered around the integration site (Figures 3a–c), yielded an on-target read depth ranging from 11 to 252x, representing 0.24%–2.28% of all reads (Figures 3a–c; Supplementary Table S2). Most samples yielded over 40x coverage, enough to reconstruct the KI consensus sequences using a *de novo* assembly approach, except for mouse #64005, which only achieved 11x coverage.

When looking at allele specificity (i.e., read counts for both wildtype and KI alleles for the three heterozygous mice (#80297, #88164, #88312), there was a slightly higher read count for the KI allele for mouse #80297, but this difference was not confirmed for the other two heterozygous mice (see Table 2), suggesting that there is no bias towards one or the other allele when performing LRS downstream of Cas9 enrichment. This may not be the case for LRS performed downstream of PCR amplification, as PCR may preferentially amplify the shorter allele.

**TABLE 2:** Read counts per allele for each heterozygous mouse sequenced in this study. Allele-specific (wildtype and knock-in) read counts for the 3 heterozygous mice sequenced in this study following Cas9 enrichment.

Mouse #	Zygosity	Transgene	Allele type	Read counts
80297	heterozygous	EGFP	wild-type	13
			knock-in	81
88164	heterozygous	EBFP2-3xFLAG	wild-type	102
			knock-in	95
88312	heterozygous	EBFP2-3xFLAG	wild-type	51
			knock-in	37

Adaptive sampling (AS) is a bioinformatic feature that enhances targeted sequencing by selectively processing reads from predefined regions of interest. As DNA strands are read, the sequence is compared to the reference sequence in real time. Should the sequences not match, the nanopore closes and rejects the DNA strand, allowing another strand to be sequenced, aiming to increase on-target yield and coverage depth (Payne et al., 2020).

To evaluate whether adaptive sampling (AS) (Martin et al., 2022) could enhance coverage post Cas9-enrichment as previously assessed (Rubben et al., 2022), two mice (#64005 and #65209) were sequenced, both with and without AS (as referenced in Table 3).

**TABLE 3:** Cas9 enrichment strategy for nanopore long read sequencing. The nanopore Cas9-targeted sequencing (nCATS) method requires enrichment of the genomic sequence of interest. This table details the sequence of the guides used to enrich specific regions centered around the transgene integration site. HOM = homozygous, HET = heterozygous, chr = chromosome.

Mouse line	hACE2		Foxg1-EGFP		Foxg1 cKI	
Mouse #	64005	65209	80297		88164	88312
HET/HOM	HOM	HOM	HET		HET	HET
Adaptive samping	No + Yes	No + Yes	No		No	No
Alignment	mm10		mm10		mm10	
Enrichment	Guide	Coordinates	Guide	Coordinates	Guide	Coordinates
sgRNA-G1	5′-CAT​GCT​GTG​CCC​CAT​TGT​GT-3′	chrX:164138258	5′-TCG​AGC​GAC​GAC​GTG​TTC​AT-3′	chr12:49385239	5′-ACA​GAT​CTC​TCT​AGC​TAG​GT-3′	chr12:49382072
sgRNA-G2	5′-TTT​AGA​ATA​AAG​CGA​AGT​AG-3′	chrX:164139210	5′-GCG​CCC​CAC​TCC​GAA​CCC​GC-3′	chr12:49384393	5′-GCA​AAG​CTC​ATC​ACG​TCG​CC-3′	chr12:49382094
sgRNA-G3	5′-GGG​CAT​CTA​TAC​TTA​TAT​TC-3′	chrX:164142052	5′-ACA​CAG​GTT​ACA​TAT​TTG​CA-3′	chr12:49386690	5′-ATG​CGG​CAT​TTG​CGC​AAC​AC-3′	chr12:49386728
sgRNA-G4	5′-AGC​TTC​TAA​CAT​TCA​AAG​GA-3	chrX:164143141	5′-CGT​CTA​TAA​ATC​ATT​ACA​AC-3′	chr12:49387509	5′-TCC​TTC​GGA​TTC​AAT​TGA​AT-3′	chr12:49386565

AS was found to increase the number of on-target reads in both cases, allowing the production of a valid consensus sequence for mouse #64005. However, the degree of improvement following AS was only moderate, with an increase in depth from 1.3 to 4.7 times (Table 4). Nonetheless, Cas9 enrichment alone yielded a sufficient number of reads (Supplementary Table S2) across most experiments for *de novo* assembly, presenting a viable option to generate consensus sequences in scenarios with limited computational resources (adaptive sampling requires a workstation equipped with a high-performance GPU). Given that standard nCATS enrichment frequently provided sufficient coverage (∼40x) for successful *de novo* assembly and reliable detection of complex events like concatemers, the added complexity and computational requirements of AS is a trade-off to consider. While we recommend considering AS if computational resources are readily available, especially for challenging targets, our results suggest that standard nCATS sequencing remains a robust and viable approach for this type of analysis when AS implementation is not feasible.

**TABLE 4:** Effect of adaptive sampling on sequencing outputs following Cas9-enriched nanopore sequencing. Details of the on-target (i.e., containing the region of interest) and off-target (i.e., not containing the region of interest) reads produced on the MinION for the same mice with and without adaptive sampling (AS). N50 = sequence length of the shortest contig at 50% of the total assembly length.

#Sample	Cas9	Adaptive sampling	On-target			Off-target		
			Total_reads	Total_base	N50	Total_reads	Total_base	N50
64005	Yes	No	11	128429	14005	4471	88654381	35125
64005	Yes	Yes	52	654184	14019	17966	10652182	494
65209	Yes	No	47	262843	5387	14313	291583183	36571
65209	Yes	Yes	61	303334	5378	19601	10280310	504

### Long read sequencing identifies occurrences of concatemerization

**FIGURE 4:**
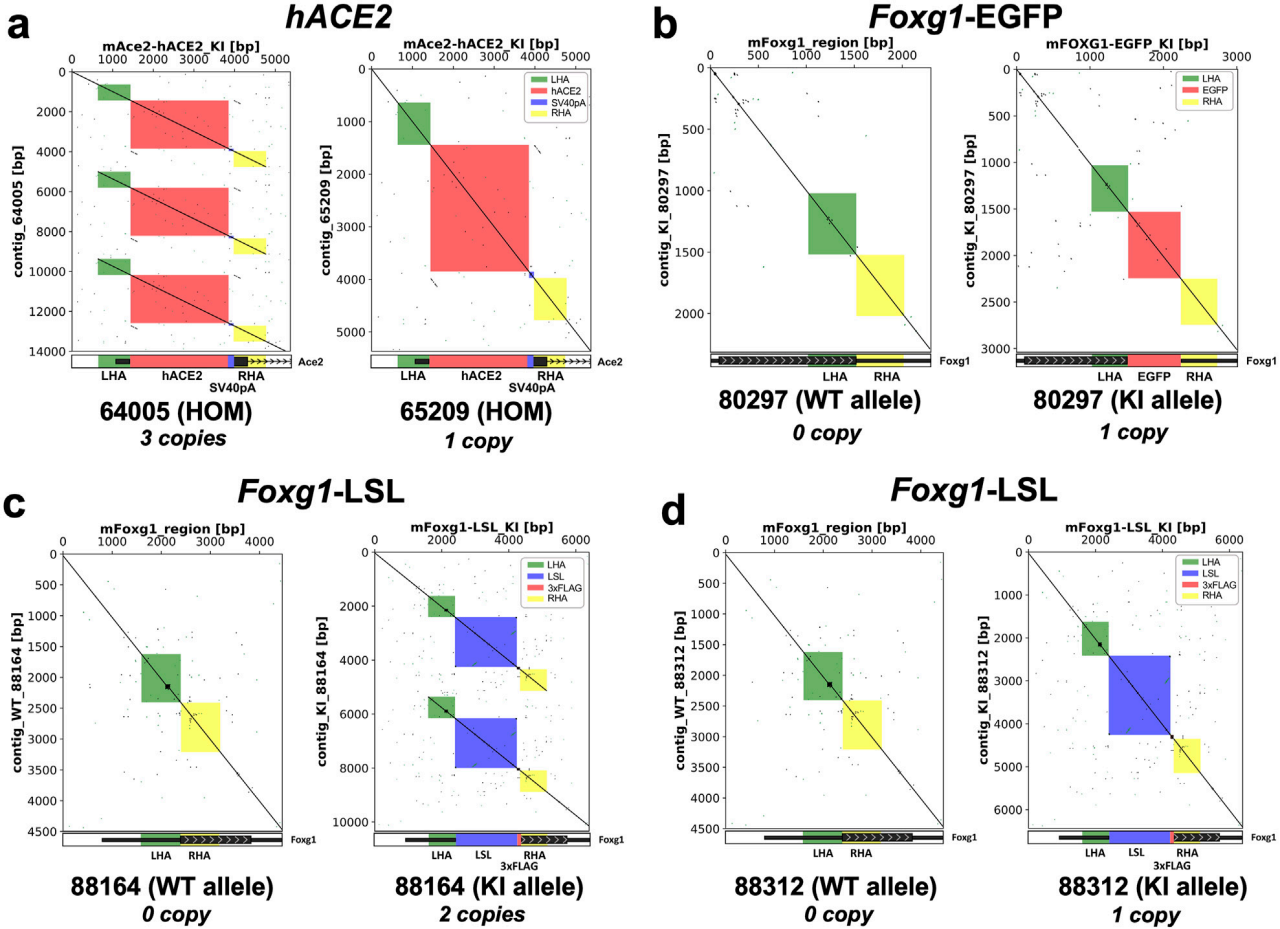
Visualization of the editing outcomes at the targeted loci following AAV-driven gene editing. Dot plots of the targeted loci show that 2 lines out of 5 (40%) carry an array of multicopies (i.e., concatemers) at the insertion site. x-axis = wildtype sequence of the targeted gene (left panels); theoretical KI sequences (right panels), y-axis = consensus sequences obtained by nanopore sequencing. A continuous line indicates full alignment, a broken line indicates a mismatch. **(a)** Dot plots analysis for the *hACE2* homozygous mice reveals that mouse #65209 carries one copy whereas mouse #64005 carries three copies of the transgene. **(b)** Dot plots analysis for the *Foxg1*-EGFP heterozygous mouse reveals that mouse #80297 carries one copy of the transgene. Dot plots analysis for the *Foxg1* cKI heterozygous mice reveals that mouse #88164 carries two copies **(c)** whereas mouse #88132 carries one copy of the transgene. **(d)** LHA = left homology arm, RHA = right homology arm, ITR = inverted terminal repeat sequence, hACE2 = human ACE2 coding sequence, SV40 pA = simian virus 40 polyadenylation signal, EGFP = Enhanced Green Fluorescence Protein sequence, 3xFLAG = triple FLAG tag, LSL = Lox-Stop-Lox.

All nCATS libraries were sequenced on either a MinION Mk1c or a GridION. The analysis of the consensus sequences was performed by aligning them to the mm10 reference assembly. Dot plots visualization (Figure 4) revealed the presence of concatemers at the insertion site.

Indeed, mouse #64005 (homozygous *hACE2*) carried three copies of the transgene (Figure 4a, left panel), while mouse #88164 (Heterozygous *Foxg1* cKI) had two copies integrated at the cut site (Figure 4c, right panel). Overall, of the five Founder lines bred forward across all gene targets, we detected concatemer integration events in two of the established lines (Table 5). Moreover, when the consensus sequences were aligned against the theoretical KI sequences (Figure 4, right panels), we did not find any mismatch, suggesting precise integration of the donor, and sequence fidelity (when aligned to the respective predicted knock-in sequence).

**TABLE 5:** Long read sequencing outcome for the selected KI mice. Details of the number of copies integrated at the insertion site for the five selected mice. Note that two out of five (40%) mice carry multicopies (i.e., concatemers).

Mouse line	hACE2		Foxg1-EGFP	Foxg1 cKI	
Mouse #	64005	65209	80297	88164	88312
HET/HOM	HOM	HOM	HET	HET	HET
Number of copies (per modified allele)	3	1	1	2	1

### Partial AAV vector sequences integration at the insertion site

**FIGURE 5:**
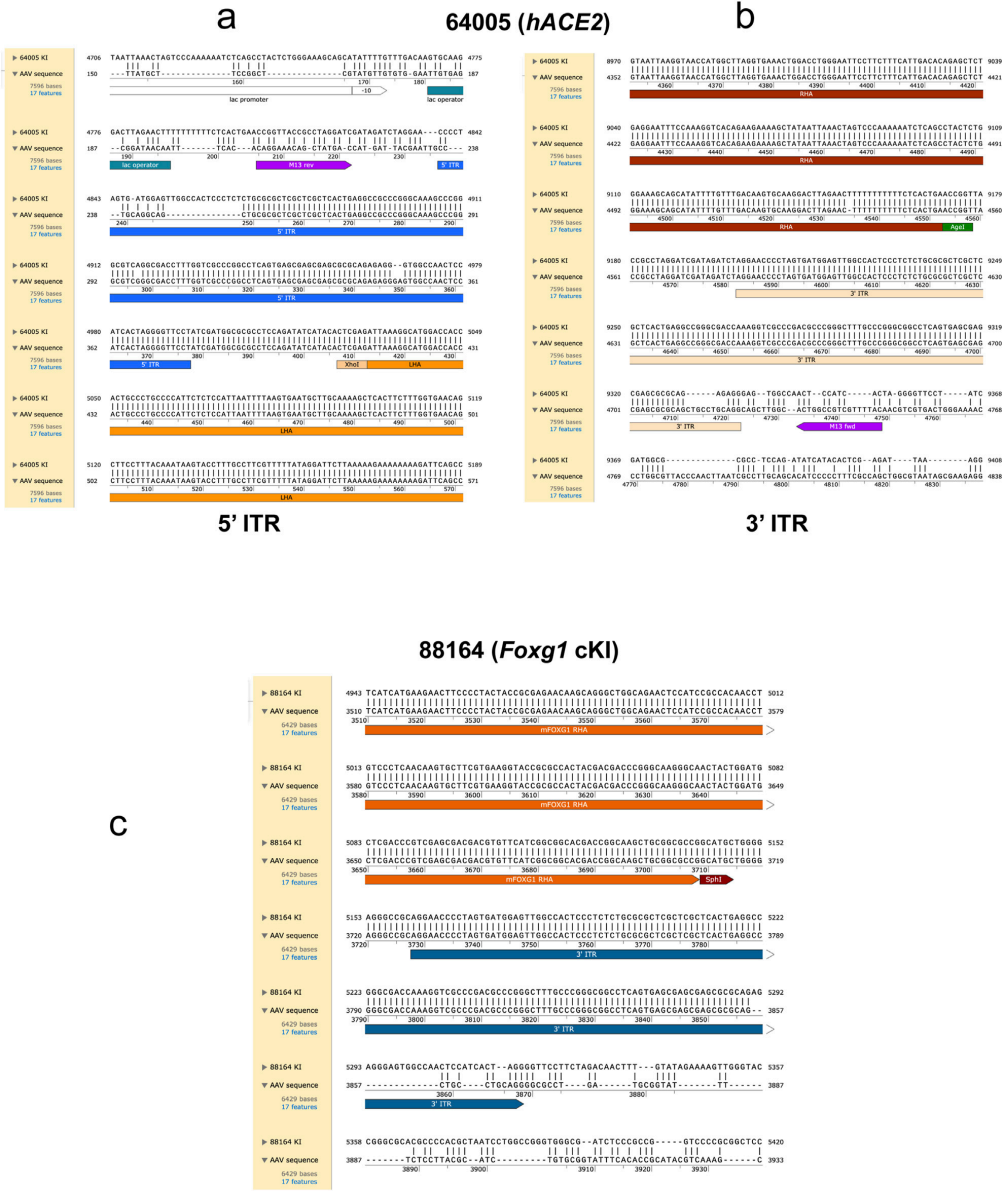
Alignment of the consensus sequences with the respective AAV vectors. The alignment identifies partial insertion of AAV vector sequences in concatemer carriers. The alignment between the *hACE2* concatemer carrier consensus sequence (mouse #64005) and the AAV vector sequences reveals that both the 5’ ITR **(a)** and the 3’ ITR **(b)** sequences align partially, illustrating the integration of these ITR sequences together with a small part of the vector containing the cloning sites (i.e., XhoI and AgeI). The alignment between the *Foxg1* cKI concatemer carrier consensus sequence (mouse #88164) and the sequence of the AAV vector used to generate this KI reveals that the 3’ ITR sequence **(c)** aligns partially, illustrating the integration of this ITR sequence together with a small part of the vector containing the cloning site (i.e., SphI). Note that the alignment of mice carrying single copies and wildtype alleles did not show any integration of the AAV vector sequences. LHA = left homology arm, RHA = right homology arm, ITR = inverted terminal repeat sequence.

**FIGURE 6:**
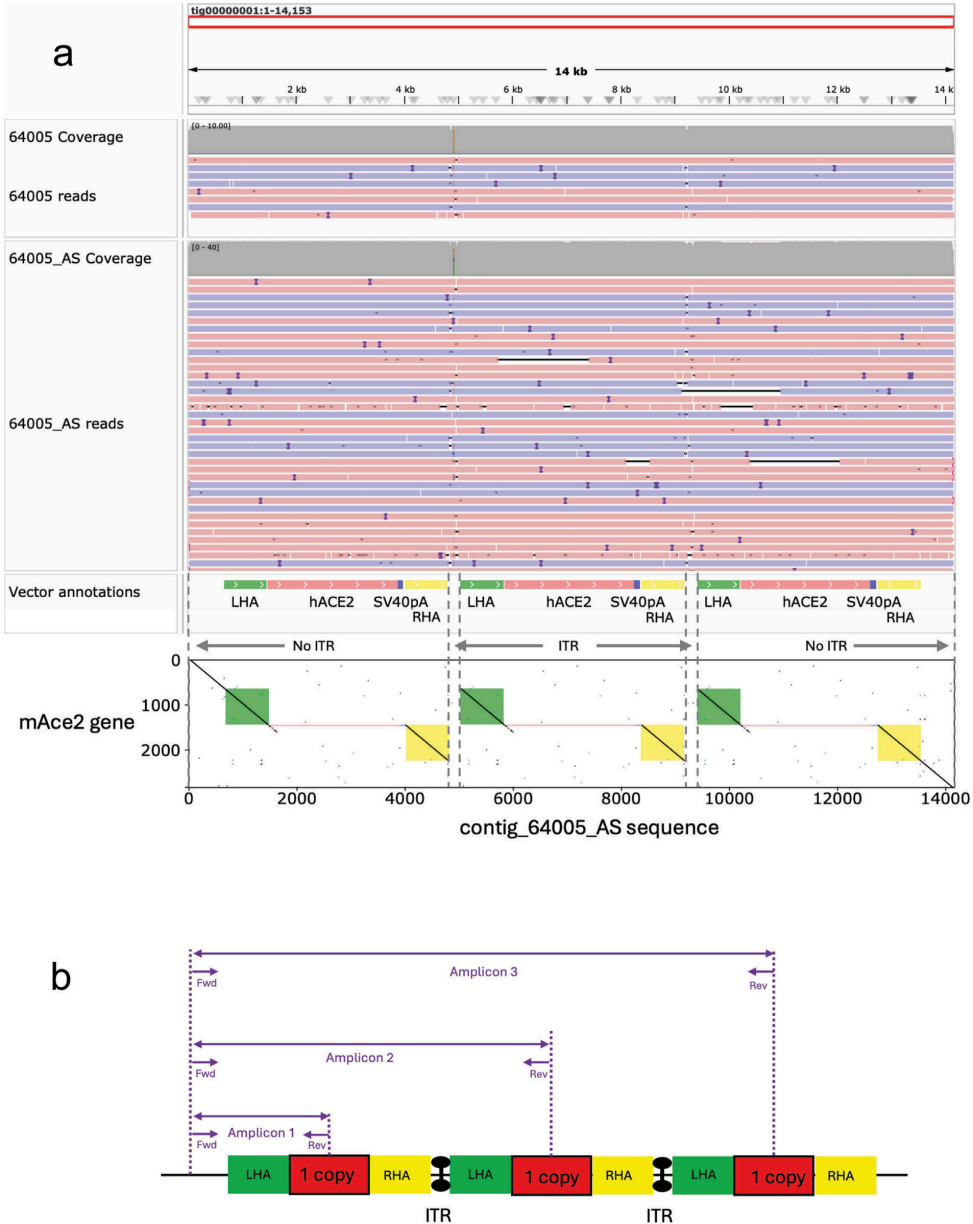
The presence of ITR sequences is only found in between copies of concatemer carriers. **(a)** Consensus sequence for the homozygous hACE2 mouse #64005 showing the location of the ITR sequences. **(b)** Schematic representation of the concatemer (3 copies) in mouse #64005 and associated 5’ junction PCR design. Note that the ITR sequences are only found at the junction of each copy, but not at the at the outermost extremities, where HDR occurred. As such, junction PCRs may not identify the presence of these ITRs, assuming that amplicons 2 and 3 may be too big to be generated.

Viral sequences were not detected in the three lines that did not have concatemer events. Conversely, both mice carrying concatemers also carried AAV vector sequences, essentially the Inverted Terminal Repeat (ITR) sequences (Figure 5). Mouse #64005 contained both ITRs (Figures 5a,b), whereas only the 3′ ITR was detected in the genome of mouse #88164 (Figure 5c). Importantly, these partial integrations were not found at the outermost extremities, where Homology Directed Repair occurred. As such, it is unlikely that the ITRs could be detected by junction PCRs, because only the biggest amplicons contain the ITR sequences, yet these amplicons are typically too big to be generated by junction PCRs (see Figure 6).

## Discussion

Gene editing in mice to generate models of human disorders is critical to biomedical research. For instance, the insertion of the human *ACE2* cDNA into its murine counterpart allows for the “humanization” of the *Ace2* gene, contributing to the development of new therapeutics against Sars-CoV-2 infections (Sun et al., 2020). Similarly, rare genetic disorders such as Foxg1 syndrome require the development of mouse models to study the pathomechanisms underlying the disorders (Younger et al., 2022).

Although the disruption of genomic sequences (knock-out) is relatively straightforward in zygotes, the targeted insertion of large transgenes following Homology Directed Repair (HDR) remains challenging. Recently, AAV-driven electroporation of CRISPR RNP complexes in mouse zygotes proved a reliable and seamless method to generate KI mice, and transgenic cores around the world routinely use this method to generate mouse models (Duddy et al., 2024).

However, quality control (QC) for these models is typically done by transgene-specific and junction PCRs, followed by Sanger sequencing. Yet this method has been shown to be inadequate to detect potential on-target mutations (Simkin et al., 2022), and leading transgenic laboratories developed guidelines for thorough validation of the alleles following gene editing (Bunton-Stasyshyn et al., 2022; Codner et al., 2018). Although off-target mutations are typically rare (Peterson et al., 2023) and no more frequent than genetic drift in mice (Peterson et al., 2023; Iyer et al., 2015; Nakajima et al., 2016), on-target mutations may also occur. It is important to note that we limited our analysis to targeted LRS, rather than whole genome sequencing, and therefore cannot rule out any potential off-target effects.

To this end, we performed Long Read Sequencing (LRS) of the targeted loci following CRISPR-READI and found occurrences of on-target illegitimate mutations. Out of five mice analyzed by LRS, two carried multicopy integration (i.e., concatemers). Furthermore, the alignment of the consensus sequences with that of the respective AAV vectors revealed partial integration of the AAV vector sequences, essentially the Inverted Terminal Repeat (ITR) sequences. Such illegitimate on-target mutations were typically not detected when we used the traditional PCR genotyping strategy (Mizuno et al., 2018), because these mutations did not occur at the outermost boundaries of the integration site. Instead, these sequences were typically found in between copies of the transgene, suggesting that the concatemerization occurred before integration. Although LRS can be performed in a multiplexed manner downstream of PCR amplification (McCabe et al., 2024), LRS downstream of Cas9 enrichment presents with the advantage of not being limited by the performance of the DNA polymerase. Indeed, long range PCRs can be challenging, and a 3 kb KI carrying 3 copies (i.e., amplicon over 10 kb, including homology arms) would hardly be amplified by PCR, even using highly performant and processive DNA polymerases. Likewise, highly repetitive or GC reach regions are often not amplifiable by PCR (see Figure 6), whereas Cas9 enrichment can be performed to sequence DNA fragments of any size and any composition.

Although concatemers may affect the expression of the transgenes at the phenotypic level, this analysis is beyond the scope of this study and has not been carried out on the KI mice.

When comparing the efficacy of Cas9 enrichment alone versus its combination with adaptive sampling, our data suggested that adaptive sampling could enhance coverage. Therefore, we advocate for the use of adaptive sampling when resources permit. Nonetheless, Cas9 enrichment alone yielded a sufficient number of reads across most experiments, presenting a viable option in scenarios with limited computational resources.

### Limitations

Although LRS downstream of Cas9 enrichment to analyze the outcome of editing events is an unbiased method that does not require PCR amplification (and as such avoids potential amplification mistakes or biases), its main limitation is its inability to be multiplexed (Scholz et al., 2024), compared to PCR-based methods (McCabe et al., 2024). Hence, because non-multiplexed analysis of the KI is substantially expensive (one mouse per flow cell), it is not possible to carry out statistical analysis on the limited number of mice (5) we sequenced. The development of a multiplexed method to run LRS downstream of Cas9 enrichment will allow for robust statistical comparison of outcome inter and intra experiments. Likewise, the limited number of mice sequenced in this study does not allow for a mechanistic analysis on the formation of concatemers and/or partial integrations. These intermolecular recombinations may originate from events such as ITR-mediated recombination (Duan et al., 1998), and/or from uncomplete quality controls during the manufacturing process of the AAV (Bai et al., 2024). Variables such as homology arm length, AAV titer, or other editing conditions may influence the frequency of concatemerization or vector integration. Further studies are required to identify the main molecular drivers of concatemerization, yet we recommend ensuring that a thorough quality control be carried out during the manufacture of AAV.

Finally, it is important to note that the High Molecular Weight (HMW) DNA used in this study was collected from the mouse kidney. A suitable HMW DNA extraction method using tail biopsies or ear notches would allow for the LRS analysis to be performed on Founder mice and avoid breeding unnecessary mice, which is a major ethical consideration. To center the analysis on the Founder mice, alternative methods such as digital droplet PCR (ddPCR) and quantitative real time PCR (qPCR) may be useful to identify copy number, and breed solely the KI mice that do not carry concatemers. Moreover, a thorough analysis of the frequency of the potential non-targeted events (both episomal presence and random integration) would inform transgenic laboratories as to the minimal number of Founders to analyze, to ensure at least one properly targeted line is generated.

### Generalization

Yet, the entire process, from DNA extraction to data analysis, was completed within 4 days. This work highlights the potential of this method to provide a rapid and efficient means of assessing transgene insertion in a timely manner. Moreover, this approach has the potential to be widely applied as a tool for identifying and characterizing structural rearrangements and repetitive regions following gene editing in mice. Although the present analysis is restricted to a limited number of mice, it has been previously demonstrated that genomic double-stranded breaks tend to capture foreign DNA (Sargent et al., 1997). As such, concatemers and/or illegitimate integrations have been observed with high frequency in different settings, including human cells edited with ZFN (Olsen et al., 2010; Radecke et al., 2010), murine embryonic stem cells edited with CRISPR (Erbs et al., 2023), *C. Elegans* (Dickinson et al., 2013) and Zebrafish (Gutierrez-Triana et al., 2018) edited with CRISPR, and cattle edited with TALEN (Norris et al., 2020). Even a typical workflow of microinjection of plasmids with CRISPR RNPs can generate up to 60% of the lines carrying multicopy integrations in mice (Skryabin et al., 2020). This could only be mitigated by biotinylation of the donor template (Medert et al., 2023). Importantly, high level of AAV vector integration (up to 47%) was also found when performing AAV-driven electroporation of CRISPR RNP complexes in cultured murine cells (Hanlon et al., 2019) and human hepatocytes (Ginn et al., 2020), in line with our findings in zygotes.

### Conclusion

Therefore, we recommend using long read sequencing as a stringent QC for KI lines generated using CRISPR-READI, and potentially other methods. This work highlights the importance of in-depth validation of the mutant lines generated by transgenic cores, which is critical to ensure reproducibility of animal research, and as such, helps prevent animal wastage. Besides, this work also yields important information with regards to the uptake of this new method, given that CRISPR-Cas9/rAAV6 therapeutic strategies are currently implemented for monogenic diseases such as severe combined immunodeficiency (SCID) (Iancu et al., 2023) while recent studies found frequent concatemeric insertions in stem cells (Suchy et al., 2024) and partial AAV vector integration after gene therapy (Simpson et al., 2023).

## Methods

### Generation of KI mouse lines

The generation of KI mice was performed following the CRISPR-READI method (Chen et al., 2019). Briefly, C57BL/6J fertilized zygotes (obtained by superovulating 3–4-week-old females with 5IU of Pregnant Mare Serum Gonadotropin and Human Chorionic Gonadotropin 46 h apart) were infected for 5 h with AAV6 carrying the respective transgene of interest in modified culture medium (Embryotech Laboratories, ETECH-EL) before thorough washing and *ex-vivo* electroporation of CRISPR reagents, as previously reported (Morey et al., 2022). As a general rule for the design of the editing strategies, we tried to select CRISPR guides (and TALENs) overlapping the insertion site, ensuring that the induced double strand break is close enough to allow for HDR to occur on the released strand, while preventing any recut following HDR. Moreover, the highest concentrations possible for each reagent were selected, while always keeping a 10 μL final volume. Recombinant AAV6 was commercially sourced (Vector Builder, pilot scale packaging >2 × 10^11^ GC/mL). The zygotes were then reimplanted into pseudopregnant outbred mice (ARC(s), Ozgene) following conventional protocols (Delerue and Ittner, 2017).

#### *hACE2* KI mice

The second exon of the *Ace2* gene (ENSEMBL ENSMUSG00000015405) was targeted using a commercially synthesised (Integrated DNA Technologies, Inc.) guide (sequence in Supplementary Table S1). This single-guide RNA (sgRNA) was rationally designed using a computational tool (Oliveros et al., 2016) to minimize off-targets (https://bioinfogp.cnb.csic.es/tools/breakingcas/) and incubated for 10 min at room temperature with *Alt-R™ S.p. Cas9* Nuclease *V3* (IDT # 1081058) to form ribonucleoprotein (RNP) complexes. TALEN mRNA (ThermoFisher Scientific) targeting the same locus was also added to the editing mix, because no suitable sgRNA could be identified to target this genomic sequence.

Following AAV infection, this editing mix was electroporated (NEPA21, Nepagene) into fertilised C57BL/6 zygotes with the respective concentrations: 200 ng/μL Cas9, 400 ng/μL sgRNA, 100 ng/μL TALEN (Table 1). For all experiments, the same electroporation parameters were used. Poring pulses: 4 pulses, 25 V, 1.5msec, 50msec intervals, 10% decay, Polarity +; Transfer pulses: 4 pulses, 3V, 50msec, 50msec intervals, 40% decay, Polarity +/−.

#### Foxg1-*EGFP* KI mice

The first exon of the *Foxg1* gene (ENSEMBL ENSMUSG00000020950) was targeted at the stop codon using two commercially synthesised (Integrated DNA Technologies, Inc.) guides (sequences in Supplementary Table S1). These single-guide RNAs (sgRNAs) were rationally designed using a computational tool (Oliveros et al., 2016) to minimize off-targets (https://bioinfogp.cnb.csic.es/tools/breakingcas/) and incubated for 10 min at room temperature with *Alt-R™ S.p. Cas9* Nuclease *V3* (IDT # 1081058) to form ribonucleoprotein (RNP) complexes.

Following AAV infection, this RNP mix was electroporated (NEPA21, Nepagene) into fertilised C57BL/6 zygotes with the respective concentrations: 200 ng/μL Cas9, 100 ng/μL each guide (Table 1).

#### Foxg1 cKI mice

These mice were generated using the exact same procedure (and concentrations) as the one used for the *Foxg1*-EGFP mice, however the sgRNAs targeted the start codon of the first exon of the *Foxg1* gene.

For all KI lines, live pups were produced and bred to establish colonies. PCR genotyping on isopropanol-precipitated DNA from tail biopsies was performed to identify potential founders. Genotyping screen consisted in transgene-specific PCR, followed by 5′ and 3′ junctions PCRs (primer sequences in Supplementary Table S1). Imaging of the electrophoresis gels was done using a BioRad Gel Doc EZ imager with default parameters. Original gels are presented in Supplementary Figure S2.

### Genomic DNA extraction

High Molecular Weight (HMW) DNA was obtained using the Monarch^®^ HMW DNA extraction kit for Tissue (T3060L, New England Biolabs), as previously described (Low et al., 2022). Briefly, fresh kidney tissues from heterozygous (HET) or Homozygous (HOM) mice were harvested, kept on ice and 20 mg of tissue was immediately processed.

### Library preparation

Enrichment of the genomic region of interest (ROI) was performed using the Cas9 Sequencing Kit (SQK-CS9109, ONT^©^) following the nCATS method (Gilpatrick et al., 2020). Briefly, following dephosphorylation, Cas9-guided adapter ligation and dA tailing were conducted on 5 μg of HMW genomic DNA for each mouse, using four rationally designed sgRNAs (Labun et al., 2016) (two upstream and two downstream of the ROI, approximately 0.7–2.4 kilobases from the insertion site (Figures 3a–c). Next, AMPure XP bead purification was performed, and final concentrations were measured using Qubit fluorometric quantification (Koetsier and Cantor, 2021) (Q33238, ThermoFisher Scientific) before loading each nCATS library on an R9.4.1 flow cell (FLO-MIN106D, ONT^©^).

### Long read sequencing

Where applicable, adaptive sampling was employed to selectively capture reads belonging to the target regions of interest (ROI) defined in the corresponding BED files (see *Supplementary Material*) specifying 15 kilobases upstream and downstream from the ROI, using the *Mus musculus* C57BL/6J (mm10) as genomic reference. Nanopore sequencing was performed with or without adaptive sampling for 72 h on a MinION Mk1b or GridION without reloading. Fast5 files and FASTQ files were collected for analysis.

### Data analysis and visualization

Data acquisition was performed using MinKNOW (v5.2.13). Base calling was executed using GUPPY (v6.4.6) with the High-accuracy Model (HAC). The resulting FASTQ files were then aligned to the mouse reference genome (mm10) using minimap2 (v2.24). The depth of reads targeting specific regions was assessed using BEDTools genomecov (v2.30). The reads that mapped to the target region encompassing sgRNAs cleavage sites were extracted using the intersect function of BEDTools (v2.30). These reads were then used for *de novo* assembly with Canu (v2.2) followed by subsequent polishing with nanopore FASTQ using two rounds of Flye-polishing (v2.9.2-b1786) and one round of Medaka-polishing (v1.8.0). Visualization of on-target reads and assembly outcomes was performed using the Integrative Genomics Viewer (IGV v2.16.2). Finally, dot plot graphs were generated using FlexiDot (v1.06) to analyse the editing outcomes at the insertion sites, while consensus sequences were aligned to the AAV vector sequences using Snapgene v7.1.1. (Figure 5).

## Data Availability

The datasets presented in this study can be found in online repositories. The names of the repository/repositories and the datasets can be found in the Supplementary Material.
