# Supplementary material for: Long read sequencing reveals transgene concatemerization and vector sequences integration following AAV-driven electroporation of CRISPR RNP complexes in mouse zygotes
Source: Front Genome Ed. 2025 Jun 4;7:1582097. doi: 10.3389/fgeed.2025.1582097 (PMC12174137; doi:10.3389/fgeed.2025.1582097)
Supplement: Supplementary file 1 [file DataSheet1.docx]

Supplementary Material Luqman et al.

# Supplementary Data

All data that support the findings of this study are available upon request. The raw sequence reads generated in this study have been submitted to the NCBI BioProject database under accession number PRJNA1076264 ([http://www.ncbi.nlm.nih.gov/bioproject/1076264](https://urldefense.com/v3/__http:/www.ncbi.nlm.nih.gov/bioproject/1076264__;!!PfbeBCCAmug!hMqWtcmcX4gzGhI07qRyX28Zq5W4ROoj8wuoCqs6Dbu6W-k06cJERjtLkVCw_prErzrtLpYm6jJbR2kIHA$)).

# Supplementary Figures and Tables

## Supplementary Tables


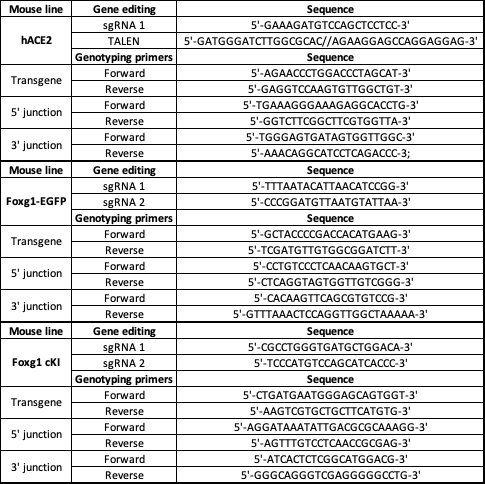


**Supplementary table S1.** **Sequences of the editing components and PCR primers used in this study**

**Supplementary table S2.** **Sequencing outputs following Cas9-enriched nanopore sequencing**

Details of the on-target (i.e., containing the region of interest), off-target (i.e., not containing the region of interest) and total reads produced on the MinION for the five selected mice. N50 = sequence length of the shortest contig at 50% of the total assembly length.

## Supplementary Figures

**
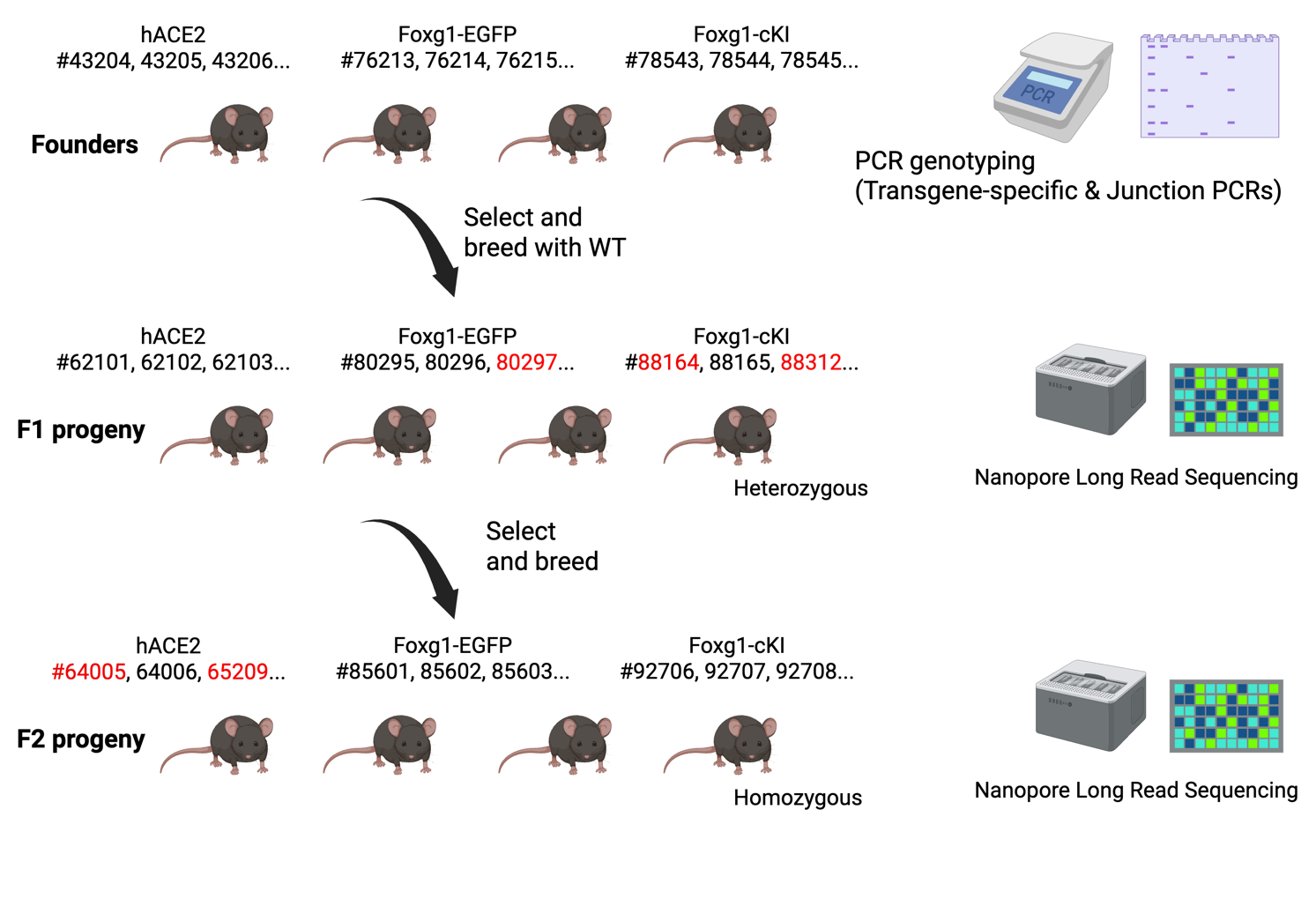
**

**Supplementary Figure 1. Workflow for the breeding, genotyping, selection, and sequencing of the mice used in this study.**

Following PCR genotyping of the Founder mice, Long Read Sequencing was performed either at the F1 or the F2 generation. Mouse numbers in red indicate selected mice that underwent LRS.

Created in BioRender. Delerue, F. (2025) https://BioRender.com/2kcss3b

**
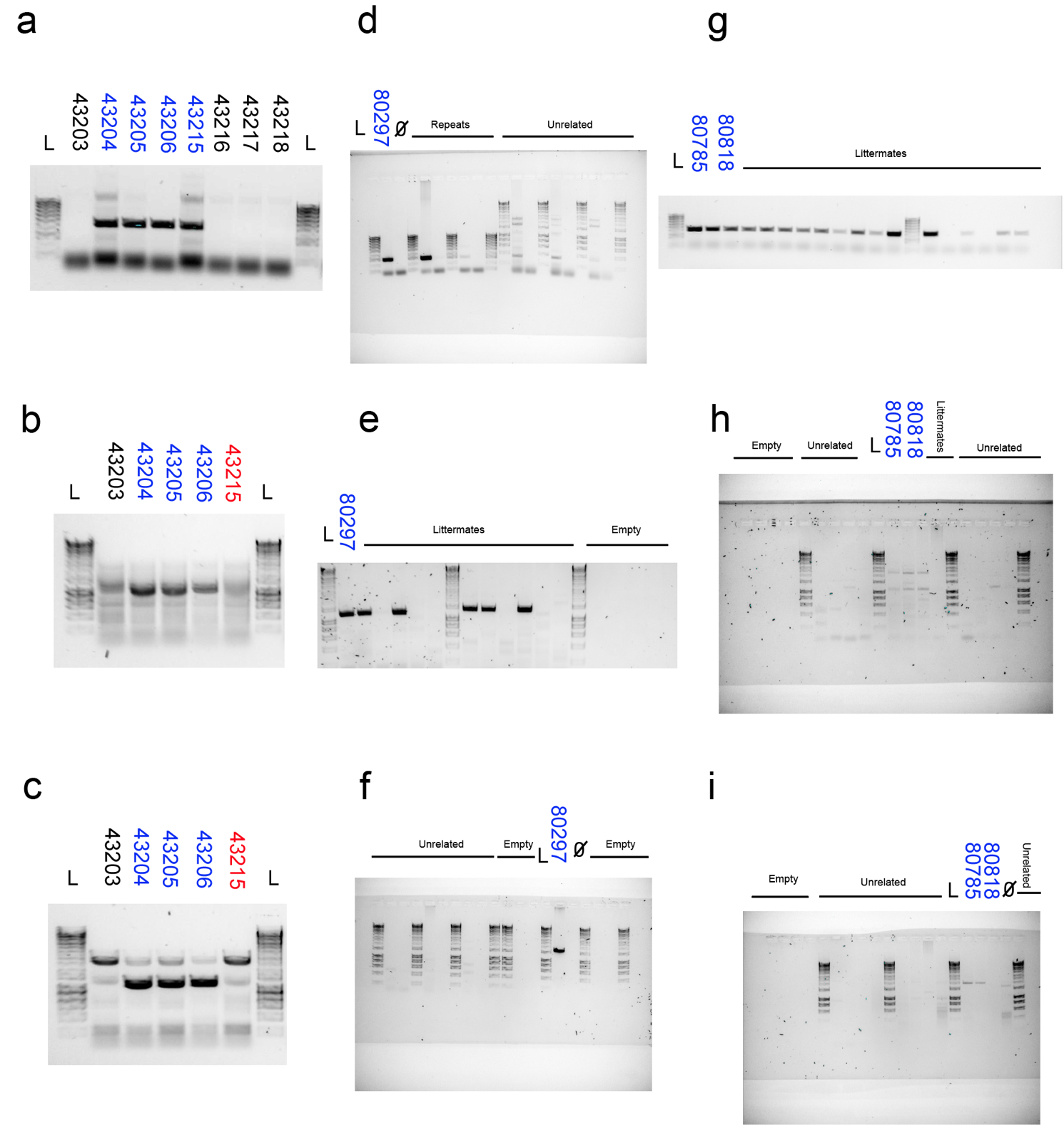
**

**Supplementary Figure 2. Original gels used in this study**

Gels are provided in the same order, position and numbering as corresponding gels in Figure 2, and where possible, uncropped.

**
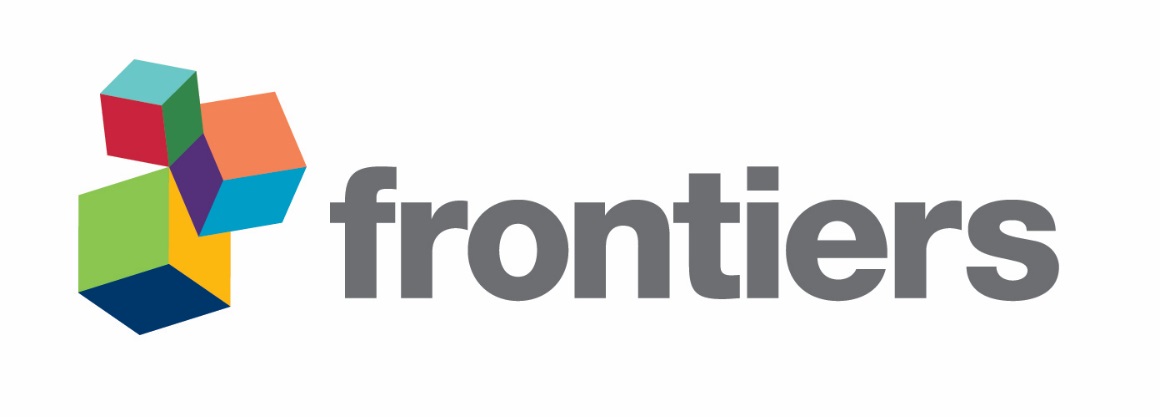
**
